# Preferred β-lactone synthesis can explain high rate of false-negative results in the detection of OXA-48-like carbapenemases

**DOI:** 10.1038/s41598-022-26735-5

**Published:** 2022-12-23

**Authors:** Vendula Studentova, Vendula Sudova, Ibrahim Bitar, Veronika Paskova, Jiri Moravec, Petr Pompach, Michael Volny, Petr Novak, Jaroslav Hrabak

**Affiliations:** 1 Biomedical Center, Faculty of Medicine in Pilsen, Charles University, Alej Svobody 76, 323 00 Pilsen, Czech Republic; 2 Department of Microbiology, Faculty of Medicine in Pilsen, Charles University, Alej Svobody 80, 323 00 Pilsen, Czech Republic; 3 Department of Clinical Biochemistry and Haematology, Faculty of Medicine in Pilsen, Charles University, Alej Svobody 80, 323 00 Pilsen, Czech Republic; 4 Institute of Microbiology of the Czech Academy of Sciences, BIOCEV, Prumyslova 595, 252 50 Vestec, Czech Republic

**Keywords:** Microbiology, Bacterial physiology

## Abstract

The resistance to carbapenems is usually mediated by enzymes hydrolyzing β-lactam ring. Recently, an alternative way of the modification of the antibiotic, a β-lactone formation by OXA-48-like enzymes, in some carbapenems was identified. We focused our study on a deep analysis of OXA-48-like-producing *Enterobacterales*, especially strains showing poor hydrolytic activity. In this study, well characterized 74 isolates of *Enterobacterales* resistant to carbapenems were used. Carbapenemase activity was determined by matrix-assisted laser desorption/ionization time-of-flight mass spectrometry (MALDI-TOF MS), liquid chromatography/mass spectrometry (LC–MS), Carba-NP test and modified Carbapenem Inactivation Method (mCIM). As meropenem-derived β-lactone possesses the same molecular weight as native meropenem (MW 383.46 g/mol), β-lactonization cannot be directly detected by MALDI-TOF MS. In the spectra, however, the peaks of m/z = 340.5 and 362.5 representing decarboxylated β-lactone and its sodium adduct were detected in 25 out of 35 OXA-48-like producers. In the rest 10 isolates, decarboxylated hydrolytic product (m/z = 358.5) and its sodium adduct (m/z = 380.5) have been detected. The peak of m/z = 362.5 was detected in 3 strains co-producing OXA-48-like and NDM-1 carbapenemases. The respective signal was identified in no strain producing class A or class B carbapenemase alone showing its specificity for OXA-48-like carbapenemases. Using LC–MS, we were able to identify meropenem-derived β-lactone directly according to the different retention time. All strains with a predominant β-lactone production showed negative results of Carba NP test. In this study, we have demonstrated that the strains producing OXA-48-like carbapenemases showing false-negative results using Carba NP test and MALDI-TOF MS preferentially produced meropenem-derived β-lactone. We also identified β-lactone-specific peak in MALDI-TOF MS spectra and demonstrated the ability of LC–MS to detect meropenem-derived β-lactone.

## Introduction

Carbapenems represent the drugs that have been efficiently used for the therapy of infections caused by Gram-negative bacteria resistant to broad-spectrum cephalosporins, i.e., those producing extended-spectrum and AmpC-type β-lactamases^1^. Degradation of carbapenems by carbapenemases, members of β-lactamases, is the most common resistance mechanism to this antibiotic class in *Enterobacterales* and non-fermenting Gram-negative bacteria. As the genes encoding for carbapenemases are usually carried on mobile genetic elements (e.g., plasmids) that can be easily transferred in microbial communities, their global spread is of utmost clinical and epidemiological importance in current medicine^2^.

As an integral part of surveillance, rapid detection of the bacteria producing carbapenemases is required^3^. Based on regional epidemiological situation, direct detection of specific enzymes, e.g., molecular-genetic detection of the genes or immunological detection, can be used^3,4^. Such assays are limited to the known carbapenemases or their variants. Therefore, they are not sufficient to detect novel or low-prevalent carbapenemases or enzymes with altered catalytic activity^3^. To eliminate that problem, carbapenemase activity can be detected directly by spectrometric assay, matrix assisted laser desorption/ionization time-of-flight mass spectrometry (MALDI-TOF MS) or pH changes of the reaction mixture^3,5–8^. All methods directly detecting changes in β-lactam ring, however, can be influenced by specific biochemical conditions of the reaction mixture required for carbapemase activity.

In the classical way of β-lactamase action, hydrolytic cleavage of the amide bond of the β-lactam ring is observed. This modification deactivates the antibiotic before it reaches its target (penicillin-binding protein). The approach of the β-lactam agent by β-lactamase is guided by relatively long-range electrostatic attractions between the carboxylate of the β-lactam and the cationic side chain of an active site amino acid in β-lactamase. A proximal residue catalytically hydrolyzes the β-lactam ring, forming an acyl enzyme complex, which is followed by further degradation^9^.

Recently, Lohans and colleagues^10,11^ published an analysis that proposed an alternative way of the modification of the β-lactam ring in some carbapenems (ertapenem, meropenem, biapenem, doripenem) by OXA-48-like enzymes, a β-lactone formation. The mechanism proposed by Lohans et al. states that the hydroxyethyl 6R side chain of carbapenem reacts with the carbonyl from the β-lactam ring to form a β-lactone. Imipenem and panipenem did not form the β-lactone ring, presumably, because the presence of a 1β methyl substituent is necessary for β-lactone formation. Lohans et al.^10^ also proposed that the methyl substituent destabilizes the conformation of the hydroxyethyl side chain required for hydrolysis of the acyl enzyme complex.

Carbapenem modification by lactonization does not change the molecular mass of the final product, therefore its identification by mass spectrometry can be difficult. We also hypothesize that β-lactone formation does not change the pH of the reaction mixture as a result of carbapenemase activity. Therefore, β-lactone formation in OXA-48-like producing bacteria could explain insufficient sensitivity of some direct methods used for carbapenemase detection reported elsewhere^12–14^. Thus, we focused our study on a deep analysis of OXA-48-like-producing *Enterobacterales*, especially strains showing poor hydrolytic activity determined by MALDI-TOF MS- and pH-based direct methods. We also identified specific peaks in MALDI-TOF MS spectra revealing meropenem-derived β-lactone.

## Materials and methods

### Bacterial isolates, susceptibility testing

Previously well characterized 74 isolates of carbapenem resistant *Enterobacterales* were used. Species identification was performed by MALDI-TOF MS—MALDI Biotyper® system (Bruker Daltonics, Bremen, Germany). In all strains, carbapenemase activity was determined by MALDI-TOF MS assay^3,12^. Carbapenemase genes were amplified and sequenced as previously described^15–19^. Minimal inhibitory concentration to 24 antibiotics including ertapenem, meropenem and imipenem was determined by broth microdilution method (Erba Lachema, Ltd., Brno, Czech Republic) and interpreted according to the criteria (version 12.0) of the European Committee on Antimicrobial Susceptibility Testing (EUCAST) (www.eucast.org). In strains used in previously published studies, whole-genome sequencing using short- and long-reads was performed^17–19^. Among them, 26 isolates produced OXA-48 carbapenemase, five isolates OXA-181 enzyme together with NDM metallo-β-lactamase, one isolate OXA-232 with co-production of NDM-1 carbapenemase, three isolates co-expressed OXA-244 and NDM-type enzymes, 26 isolates produced KPC-type carbapenemase, 6 isolates produced a metallo-β-lactamase (NDM, VIM) and 8 isolates expressed low-prevalent carbapenemases (GES, IMI). As a negative control, *Escherichia coli* ATCC14169 was used. Detailed characterization of the strains is summarized in Table 1.

**Table 1:** Characteristics of bacterial isolates used in the study.

Strain number	Species	OXA-48-like carbapenemase	Other beta-lactamases	Carba NP	mCIM (inhibition zone diameter [mm])	β-lactone (*m/z* 362.5)	MIC [mg/L]			References
							ERT	MER	IMI	
ATCC14169 (negative control)	*Escherichia coli*	–	NT	**−**	30	−	≤ 0.125	≤ 0.125	≤ 0.125	**–**
V117 (positive control)	*Klebsiella pneumoniae*	–	KPC-2, TEM-1	+	NZ	−	> 16	> 16	> 16	^16^
P370	*Enterobacter cloacae*	OXA-48	NT	**−**	NZ	+	0.5	0.125	0.25	This study
P759	*Klebsiella pneumoniae*	OXA-48	NT	+	NZ	−	> 16	2	4	This study
4292	*Enterobacter cloacae*	OXA-48	CTX-M-15, OXA-1, TEM-1	**−**	NZ	+	> 16	0.5	4	^17^
C0417 646/2	*Escherichia coli*	OXA-48		**−**	NZ	+	2	0.125	0.5	^17^
32005	*Escherichia coli*	OXA-48	TEM-1	+	21	+	1	0.125	0.25	^17^
C 0426 031	*Escherichia coli*	OXA-48		+	24	+	0.25	0.125	1	^17^
C 374 996	*Klebsiella pneumoniae*	OXA-48		+	NZ	**−**	2	1	2	^17^
C 0417 153	*Klebsiella pneumoniae*	OXA-48		+	NZ	**−**	4	0.25	0.5	^17^
C 381 700/1	*Klebsiella pneumoniae*	OXA-48	TEM-1	**−**	NZ	**−**	2	1	1	^17^
D 382 929	*Klebsiella pneumoniae*	OXA-48	CTX-M-14	**−**	NZ	+	1	≤ 0.125	2	^17^
4976	*Klebsiella pneumoniae*	OXA-48		**−**	NZ	+	4	0.25	2	^17^
4963	*Klebsiella pneumoniae*	OXA-48	CTX-M-15, OXA-1, TEM-1	**−**	NZ	+	> 16	8	> 16	^17^
29097	*Klebsiella pneumoniae*	OXA-48		**−**	NZ	+	2	0.25	1	^17^
5159	*Klebsiella pneumoniae*	OXA-48	CTX-M-15, OXA-1, TEM-1	**−**	NZ	+	> 16	16	> 16	^17^
C 0418 921/2	*Klebsiella pneumoniae*	OXA-48	CTX-M-15, TEM-1	**−**	NZ	+	4	0.25	0.5	^17^
30715	*Klebsiella pneumoniae*	OXA-48	CTX-M-15	**−**	NZ	+	> 16	> 16	1	^17^
30891	*Klebsiella pneumoniae*	OXA-48	CTX-M-15, TEM-15	**−**	NZ	+	4	0.5	1	^17^
30890	*Klebsiella pneumoniae*	OXA-48	CTX-M-15	**−**	NZ	+	2	1	2	^17^
C 420 382	*Klebsiella pneumoniae*	OXA-48	CTX-M-15, TEM-1	**−**	NZ	+	4	0.25	2	^17^
31329	*Klebsiella pneumoniae*	OXA-48	CTX-M-15,OXA-1, TEM-1	**−**	NZ	+	2	1	> 16	^17^
C 423 495	*Klebsiella pneumoniae*	OXA-48	CTX-M-15, TEM-1	**−**	NZ	+	2	0.25	2	^17^
29144	*Klebsiella pneumoniae*	OXA-48	CTX-M-15, OXA-1, TEM-1	+	24	+	> 16	> 16	> 16	^17^
C 423 770/3	*Klebsiella pneumoniae*	OXA-48	CTX-M-15, TEM-1	+	25	+	> 16	8	> 16	^17^
C 423 482	*Klebsiella pneumoniae*	OXA-48	CTX-M-15, TEM-1	+	15	+	2	0.25	2	^17^
C 424 100	*Klebsiella pneumoniae*	OXA-48	CTX-M-15, TEM-1	+	22	+	2	0.25	2	^17^
31569	*Klebsiella pneumoniae*	OXA-48	CTX-M-15, TEM-1	+	24	+	> 16	8	2	^17^
trf 47 733-3	*Escherichia coli*	OXA-181	NDM-5	+	NZ	−	> 2	4	2	^19^
Vit-2	*Escherichia coli*	OXA-181	NDM-5	+	NZ	−	> 2	> 16	8	^20^
trf 50 595	*Escherichia coli*	OXA-181	NDM-1	+	NZ	+	1	≤ 0.125	0.5	^19^
47733	*Klebsiella pneumoniae*	OXA-181	NDM-5, CTX-M-15, SHV-11	+	18	−	> 2	> 16	> 16	^19^
50595	*Klebsiella pneumoniae*	OXA-181	NDM-1	+	NZ	−	> 2	16	8	^19^
30929	*Klebsiella pneumoniae*	OXA-232	NDM-1, CTX-M-15, OXA-1	+	NZ	+	> 16	8	> 16	^17^
52148	*Escherichia coli*	OXA-244	NDM-5	+	NZ	−	> 2	4	8	^19^
trf 52 148-1	*Escherichia coli*	OXA-244	NDM-5	+	NZ	−	> 2	4	2	^19^
51015	*Klebsiella pneumoniae*	OXA-244	NDM-1	+	NZ	+	> > 2	8	> 16	^19^
48846	*Citrobacter freundii*	–	KPC-3	+	NZ	**−**	> 2	8	8	^18^
49141	*Citrobacter freundii*	–	KPC-3	+	NZ	**−**	> 2	8	8	^18^
49969	*Citrobacter freundii*	–	KPC-3	+	NZ	**−**	> 2	8	8	^18^
50935	*Citrobacter freundii*	–	KPC-3	+	NZ	**−**	> 2	8	8	^18^
36567	*Enterobacter aerogenes*	–	IMI-2	+	17	**−**	> 2	> 16	> 16	^21^
46506	*Enterobacter aerogenes*	–	KPC-3	+	NZ	**−**	> 2	16	16	^18^
54863	*Enterobacter asburiae*	–	GES-5, TEM-1, OXA-1	+	22	**−**	> 2	> 16	> 16	^22^
35771	*Enterobacter cloacae*	–	GES-5	+	25	**−**	8	> 16	> 16	^22^
54862	*Enterobacter cloacae*	–	GES-5, TEM-1	+	24	**−**	> 2	16	4	^22^
48293	*Enterobacter hormaechei*	–	KPC-3	+	NZ	**−**	> 2	8	16	^18^
49583	*Enterobacter hormaechei*	–	KPC-3	+	NZ	**−**	> 2	16	> 16	^18^
trf 35 771-3	*Escherichia coli*	–	GES-5	+	26	**−**	> 2	> 16	> 16	^22^
trc 36 567; -3; IV	*Escherichia coli*	–	IMI-2	+	NZ	**−**	> 2	8	> 16	^21^
53083	*Escherichia coli*	–	KPC-3	+	NZ	**−**	> 2	8	4	^18^
Vit-1	*Escherichia coli*	–	NDM-5	+	NZ	**−**	> 2	> 16	16	^20^
45182	*Klebsiella michiganensis*	–	KPC-3	+	NZ	**−**	> 2	8	8	^18^
I 0879 558	*Klebsiella oxytoca*	–	GES-7	+	NZ	**−**	> 2	8	2	^23^
V 431	*Klebsiella pneumoniae*	–	VIM-1	+	NZ	**−**	0.125	4	8	^24^
V 476	*Klebsiella pneumoniae*	–	VIM-1	+	NZ	**−**	2	8	2	^24^
Lar-38i	*Klebsiella pneumoniae*	–	VIM-1, NDM-1	+	NZ	**−**	> 8	> 16	8	^25^
Lar-61	*Klebsiella pneumoniae*	–	VIM-1, NDM-1	+	NZ	**−**	> 8	> 16	8	^25^
Lar-62i	*Klebsiella pneumoniae*	–	VIM-1, NDM-1	+	NZ	**−**	> 8	> 16	8	^25^
A 9853	*Klebsiella pneumoniae*	–	KPC-3	+	NZ	**−**	> 2	> 16	> 16	^18^
47693	*Klebsiella pneumoniae*	–	KPC-3	+	20	**−**	> 2	> 16	> 16	^18^
51069	*Klebsiella pneumoniae*	–	KPC-3	+	NZ	**−**	> 2	> 16	> 16	^18^
A 4411	*Klebsiella pneumoniae*	–	KPC-3	+	NZ	**−**	> 2	> 16	16	^18^
51248	*Klebsiella pneumoniae*	–	KPC-3	+	NZ	**−**	> 2	> 16	> 16	^18^
51483	*Klebsiella pneumoniae*	–	KPC-3	+	NZ	**−**	> 2	16	8	^18^
52810	*Klebsiella pneumoniae*	–	KPC-3	+	NZ	**−**	> 2	16	16	^18^
52813	*Klebsiella pneumoniae*	–	KPC-3	+	NZ	**−**	> 2	> 16	16	^18^
46903	*Morganella morganii*	–	KPC-3	+	NZ	**−**	> 2	2	8	^18^
48659	*Morganella morganii*	–	KPC-3	+	NZ	**−**	> 2	2	16	^18^
50821	*Morganella morganii*	–	KPC-3	+	NZ	**−**	> 2	2	8	^18^
51087	*Morganella morganii*	–	KPC-3	+	NZ	**−**	> 2	8	16	^18^
52808	*Proteus mirabilis*	–	KPC-3	−	NZ	**−**	> 2	2	> 16	^18^
52260	*Proteus mirabilis*	–	KPC-3	+	NZ	**−**	> 2	4	> 16	^18^
52812	*Proteus mirabilis*	–	KPC-3	+	NZ	**−**	> 2	4	> 16	^18^
53415	*Proteus mirabilis*	–	KPC-3	+	NZ	**−**	> 2	4	> 16	^18^

*NT* not tested, *NZ* no inhibition zone detected, *ERT* ertapenem, *MER* meropenem, *IMI* imipenem.

### Carbapenemase detection by MALDI-TOF MS analysis, Carba NP test, and modified carbapenem inactivation method (CIM)

Carbapenemase activity was detected by the MALDI-TOF MS meropenem hydrolysis assay, Carba NP test, and modified Carbapenem Inactivation Method (mCIM) as previously described^26–28^. For all tests, KPC-2-producing *Klebsiella pneumoniae* ST258 and *Escherichia coli* ATCC14169 were used as a positive and negative control respectively. The bacteria were cultivated on Mueller–Hinton agar overnight. Statistical comparison of the methods was performed by Fisher’s exact test.

### Carbapenemase detection by MALDI-TOF MS analysis

Carbapenemase activity was detected by the MALDI-TOF MS meropenem hydrolysis assay as previously described^26^.

Briefly, the bacteria were suspended in 20 mM Tris–HCl + 20 mM NaCl, pH 7.0 (Sigma-Aldrich, Prague, Czech Republic), to a density equivalent to a 3.0 McFarland standard. The 1-ml aliquot of the suspension was centrifuged; the pellet was resuspended in 50 μl of a reaction buffer (20 mM Tris–HCl, 0.01% sodium dodecyl sulfate, pH 7.0; Sigma-Aldrich), supplemented with 0.1 mM meropenem (Astra Zeneca, Macclesfield, United Kingdom) and with or without 50 mM ammonium bicarbonate (NH_4_HCO_3_). After incubation at 35 °C for 2 h, the reaction mixture was centrifuged and 1 μl of the supernatant was applied on MALDI target, allowed to dry and overlayed by 1 μl of 10 mg/l of dihydroxybenzoic acid solution (DHB) dissolved in 50% ethanol (Sigma-Aldrich, Prague, Czech Republic) and allowed to dry on a target. Spectra were measured within the *m/z* range 300–500 using a microflex LT and rapifleX mass spectrometers (Bruker Daltonics, Bremen, Germany) and analyzed by the flexAnalysis 4.0 software.

### Carbapenemase detection by Carba NP test

Carba NP test was prepared as previously described^27^. A full 10 μl inoculation loop of culture was resuspended in 100 µL lysis buffer B-PERII (Thermo Scientific Pierce, Rockford, IL, USA) in two Eppendorf tubes and vortexed. Then, 100 µL of a reaction solution and 100 µL of imipenem monohydrate containing phenol red (all chemicals were obtained from Sigma Aldrich, Prague, Czech Republic) were added. Incubation was performed at 35 °C for 3 h. The results were independently interpreted by two technicians. The absorbance was simultaneously measured at 580 nm using Synergy H1 spectrophotometer (Agilent Biotek, CA, USA).

### Carbapenemase detection by modified carbapenem inactivation method (mCIM)

For the mCIM^28^, a full 10 μl inoculation loop of culture was resuspended in 1 ml of Tryptic Soy Broth followed by the immersion of 10 μg meropenem disk (Bio-Rad, Prague, Czech Republic) and incubated at 35 °C for 4 h. After incubation, the disk was placed on Mueller–Hinton agar plate inoculated by *Escherichia coli* ATCC 29522. After overnight cultivation, inhibition zones were measured. The mCIM test was interpreted as positive if the inhibition zone diameter was < 19 mm.

### Carbapenemase activity in H_2_^18^O

To demonstrate incorporation of a water molecule into meropenem degradation products, the reaction was performed in an isotopically modified water H_2_^18^O. The reaction mixture was prepared by mixing of 2.5 μL of 1 M Tris–HCl, pH 7.0, and 2.5 μL of 1 mM meropenem dissolved in non-isotopically modified water, and 250 μL of H_2_^18^O (Merck KGaA, Darmstadt, Germany). An overnight bacterial culture cultivated on Mueller–Hinton agar at 35 °C was suspended in 20 mM Tris–HCl plus 20 mM NaCl, pH 7.0 (Merck KGaA, Darmstadt, Germany) to a density equivalent to a 3.0 McFarland standard. As controls, we used KPC-2-producing *Klebsiella pneumoniae* ST258 and *Escherichia coli* ATCC 14,169. The 1-ml aliquot of the suspension was centrifuged, and the pellet was resuspended in 50 μl of a reaction buffer containing isotopically modified water. The reaction was incubated at 35 °C for 3 h, centrifuged and the supernatant was used for subsequent MALDI-TOF MS and LC–MS measurements.

### Liquid chromatography/mass spectrometry analysis (LC–MS)-ion trap mass spectrometry

Fifty µL of the supernatant of the reaction mixture prepared as described above was mixed with 150 µL 0.1% formic acid, 10 µL ultrapure water and 790 µL acetonitrile. The mixture was filtered through PTFE syringe filters (0.22 µm) into HPLC vials. The analysis was performed on ion trap AmazonSL mass spectrometer (Bruker, Bremen, Germany) after separation of the sample on Dionex UltiMate 3000 UHPLC—Standard (Thermo Fisher Scientific, Waltham, Massachusetts, USA) with AQUITY UPLC BEH Amide Column (130 Å, 1.7 µm, 2.1 mm × 100 mm fitted with precolumn AQUITY UPLC BEH Amide VanGuard Pre-column 130 Å, 1.7 µm, 2.1 × 5 mm (Waters, Milford, Massachusetts, USA). Isocratic elution was used with a mobile phase consisting of 80% of acetonitrile and 20% of 5 mM ammonium formate buffer (pH 5.6). Total run time was 7 min at a flow rate 0.4 mL/min. Injection volume was 10 µL. The ion source temperature and desolvation temperature was set to 180 °C. Nitrogen was used as a nebulizer (4.0 L/min, 7.3 psi) and desolvation gas. Helium (purity 5.0) was used as a collision gas and set at 3.5010^–6^ mbar. Capillary voltage was set to 5000 V. Quantification was performed in the multiple reaction monitoring (MRM) mode (Scan mode: enhanced resolution). Spectra were analyzed by DataAnalysis 4.2 (Bruker Daltonik, Bremen, Germany).

### Liquid chromatography/mass spectrometry analysis (LC–MS)–high resolution mass spectrometry

The supernatants of the reaction mixture were injected and subsequently desalted on-line using reverse-phase microtrap column (Luna Omega 5um Polar C18, 100 Å, 20 × 0.3 mm, Phenomenex). The analytes eluted from the microtrap were separated on the reverse-phase analytical column (Luna Omega 3um Polar C18, 100 Å, 150 × 0.3 mm, Phenomenex) at 50 °C using Agilent 1290 Infinity II UHPLC system at flow rate 10 µl/min in water/acetonitrile gradient (mobile phase [A] was 98% water and 2% acetonitrile with 0.1% formic acid; mobile phase [B] was 98% acetonitrile and 2% water with 0.1% formic acid; the gradient started at 5% [B] and reached 90% [B] in 30 min). The eluted analytes were analyzed by 15 T solariX XR FT-ICR mass spectrometer (Bruker Daltonics). Mass spectral data were collected in positive broadband mode over the m/z range 150–1500, with 1 M data points transient and 0.2 s ion accumulation with two averaged scans per spectrum. Data acquisition and data processing were performed using ftmsControl 2.1.0 and DataAnalysis 5.0.

## Results

### Biochemical analysis

For the initial experiments, two OXA-48-producing strains with different MICs' patterns were selected. Using MALDI-TOF MS analysis, the strain Nr. P759 (ertapenem MIC > 16 mg/L, meropenem MIC ≈ 2 mg/L, imipenem MIC ≈ 4 mg/L) showed standard hydrolysis, i.e., presence of decarboxylated degradation product (m/z = 358.5) and its sodium adduct (m/z = 380.5). The strain Nr. P370 was susceptible to ertapenem, meropenem and imipenem (ertapenem MIC≈0.5 mg/L, meropenem MIC ≈ 0.125 mg/L, imipenem MIC ≈ 0.25 mg/L) with very weak meropenem hydrolysis, i.e., presence of a peak at m/z = 384.5 representing intact meropenem with low intensity peaks representing hydrolyzed products in the mass spectra.

As the meropenem-derived β-lactone has the same molecular weight as a native meropenem molecule (MW 383.46 g/mol), we hypothesized that both isomers can be resolved chromatographically and detected by mass spectrometry at different retention times. This was confirmed using ion trap mass spectrometer coupled to an analytical flow LC. The data in Fig. S1A show two different chromatographically resolved peaks that both corresponds to nominal m/z = 384. This demonstrates that exposure of meropenem to strain P370 creates a species isomeric with meropenem. The data obtained with strain P759 (Fig. S1B) show no peak that would correspond to m/z = 384, but a decarboxylated degradation product at m/z = 358, which represents the classical way of meropenem inactivation.

**Figure 1:**
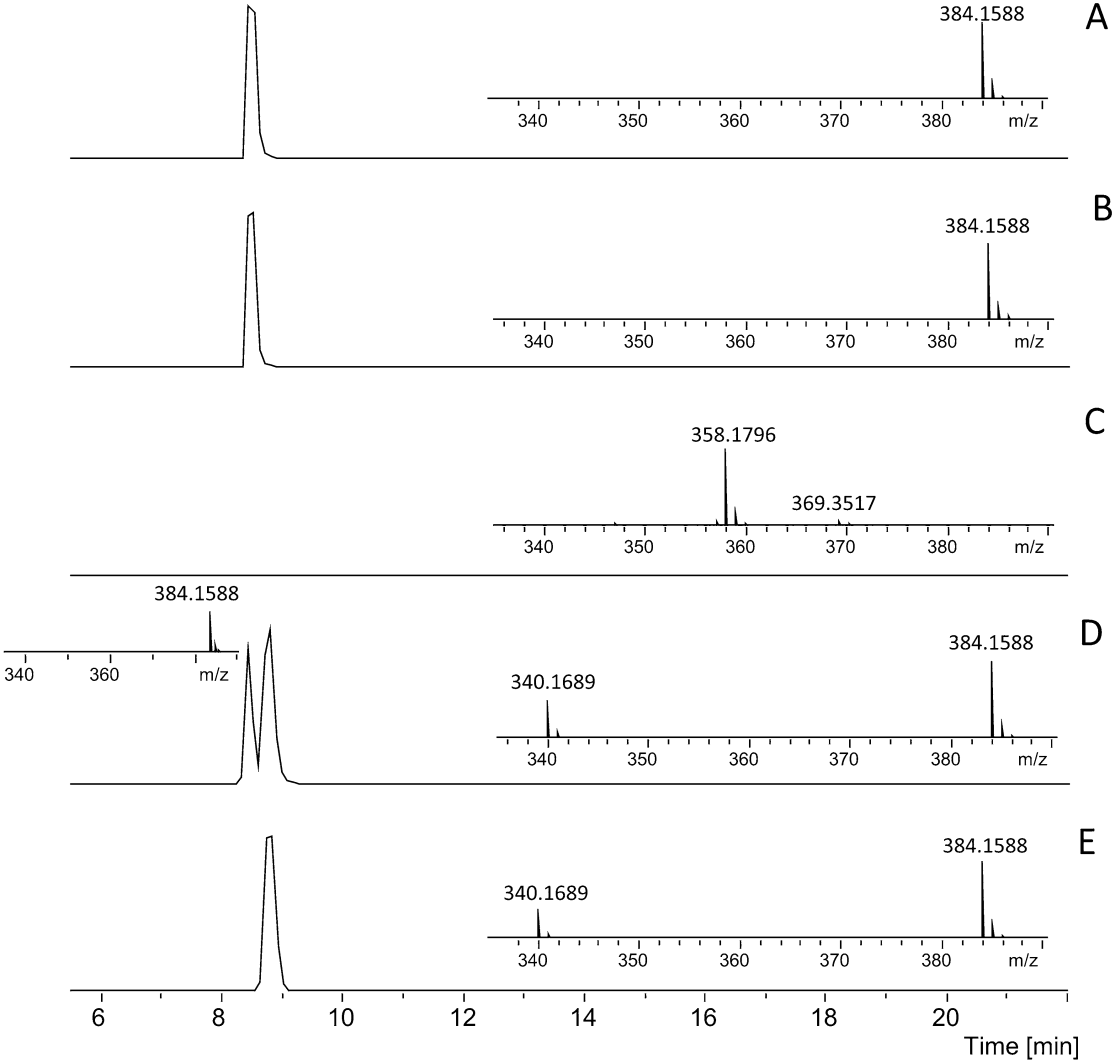
LC–MS analysis of meropenen before and after exposure to the different OXA-48 strains. Extracted Ion Chromatograms (EIC) of m/z = 384.15 (± 0.2Th) show retention behavior of meropenem and meropenem-derived β-lactone isomer. Insets show mass spectra from the apex of each chromatographic peak. (**A**) EIC of pure meropenem standard; (**B**) negative control, EIC of meropenem exposed only to *E. coli* ATCC 14,169; (**C**) positive control, EIC of meropenem exposed to strain P759. There is no chromatographic peak corresponding to meropenem, the inset mass spectrum shows degradation product at m/z = 358 at the retention time of meropenem standard; (**D**) meropenem exposed to strain P370 and partially converted to β-lactone isomer; EIC shows chromatographic resolution of original meropenem (earlier peak) and newly formed β-lactone isomer of meropenem (later peak). Inset mass spectrum for the early peak is identical with the standard meropenem spectrum and with the negative control spectrum, while mass spectrum of newly formed isomer also contains peak at m/z = 340; (**E**) meropenem exposed to strain P370 and fully converted to the β-lactone, as can be seen from the shift in retention time. Mass spectrum contains peaks at m/z = 340 and 384.

The identification of the molecules was first confirmed by analyzing the samples using high-resolution accurate mass (HRAM) FTICR instrument. Figure 1 shows LC–MS analysis of meropenem subjected to incubation with different strains. Both detected molecules (meropenem and its suspected β-lactone isomer) correspond to the theoretical molecular formula of protonated meropenem (C_17_H_26_O_5_N_3_S) with mass error of 0.05 ppm (50 ppb) and are identical in terms of molecular mass. The later eluting isomer is accompanied by a peak at *m/z* = 340.1689, indicating decarboxylation (with mass accuracy of 0.05 ppm). Figure 1A shows analysis of pure meropenem standard. Exposing meropenem only to *E. coli* ATCC 14169 (Fig. 1B) leads to the identical LC–MS results, as this strain does not transform meropenem molecule in any way. In the strain P759 (Fig. 1C) the meropenem molecule completely degraded and no peak that would correspond to m/z = 384 was detected. Exposure of meropenem sample to strain P370 results in different degradation process. Figure 1D shows a partial and Fig. 1E a complete conversion of meropenem. The peak of meropenem converted to β-lactone isomer elutes later and chromatographically separates from intact meropenem. The mass spectrum of meropenem isomer obtained from the apex of the chromatographic peak also includes a new peak at m/z = 340, which corresponds to decarboxylated meropenem. This decarboxylated species is not detected in the mass spectrum from the apex of meropenem standard chromatographic peak.

To confirm identification, a sample fully converted by the strain P370 was spiked with the original meropenem standard. Figure S2 shows the same chromatographic behavior, with spiked meropenem standard eluting first and not showing the peak at m/z = 340. Using the same spiked sample, Collision Induced Dissociation (CID) was performed to obtain MSMS spectra of both isomeric protonated ions at m/z = 384 and the decarboxylated ion at m/z = 340 (Fig. S2). Fragmentation spectra of both peaks at m/z = 384 clearly show different patterns. Spectrum of the first isomer (intact meropenem) is dominated by fragment peak at m/z = 141, which corresponds to cleavage of the C-S bond in meropenem, as reported previously^29^. The converted isomer MSMS spectrum is dominated by a base peak at m/z = 175 and does not contain the diagnostic meropenem peak at m/z = 141. Both isomeric precursors provide minor decarboxylated fragment ion at m/z = 340. Fragmentation of precursor at m/z = 340, which coelutes together with converted isomer, also provides a fragment peak at m/z = 175 (Fig. S3), which indicates that the ion at m/z = 340 is structurally related to the converted isomer of meropenem.

### MALDI-TOF mass spectrometry

**Figure 2:**
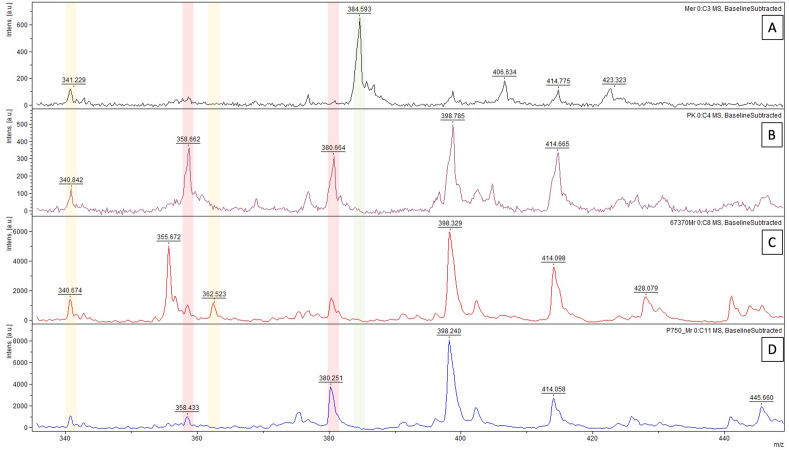
MALDI-TOF MS analysis of carbapenemase activity. Panel (**A**) shows a native meropenem (m/z = 384.5) and its sodium adduct (m/z = 406.5). Panel (**B**) shows a positive control—KPC-2-producing *Klebsiella pneumoniae* ST258. Panel (**C**) represents the sample P370 with a visible sodium adduct of meropenem-derived β-lactone (m/z = 362.5). Panel (**D**) shows the strain P759 with decarboxylated hydrolyzed meropenem (m/z = 358.5) and its sodium adduct (m/z = 380.5).

By MALDI-TOF mass spectrometry, we identified a peak of m/z = 362.5 representing sodium adduct of decarboxylated β-lactone (Fig. 2). The peak was identified in 22 OXA-48 producing bacteria, and one OXA-181-, one OXA-232- and one OXA-242-produing isolates co-expressing NDM metallo-β-lactamase. In the other six OXA-48-like producing bacteria co-producing NDM-type carbapenemase, efficient hydrolytic activity demonstrated by the identification of hydrolytic products (m/z = 358.5 and 380.5) has been identified. No difference was observed after reaction in the buffer containing NH_4_HCO_3_ or without. In the strains producing class A or/and class B carbapenemases or carbapenemase-non-producing bacteria, no peak at m/z = 362.5 was detected. In all the spectra, including spectra of native meropenem, a peak of m/z = 340.5 was identified at a low intensity. This peak represents a meropenem molecule decarboxylated at a position 3. In OXA-48 producing bacteria showing β-lactone formation, the intensity of the mentioned peak increased to an equivalent of the β-lactone sodium adduct (m/z = 362.5).

### Confirmation of carbapenemase activity in H_2_^18^O

Using isotopically modified water, we identified a peak at m/z = 360.5 representing decarboxylated meropenem hydrolytic product with incorporated ^18^O in the strain P759 as well as in a positive control by MALDI-TOF MS as well as LC–MS analysis. The peak at m/z = 382.5 representing the decarboxylated sodium adduct of meropenem with incorporated ^18^O was observed in MALDI-TOF MS analysis only.

**Figure 3:**
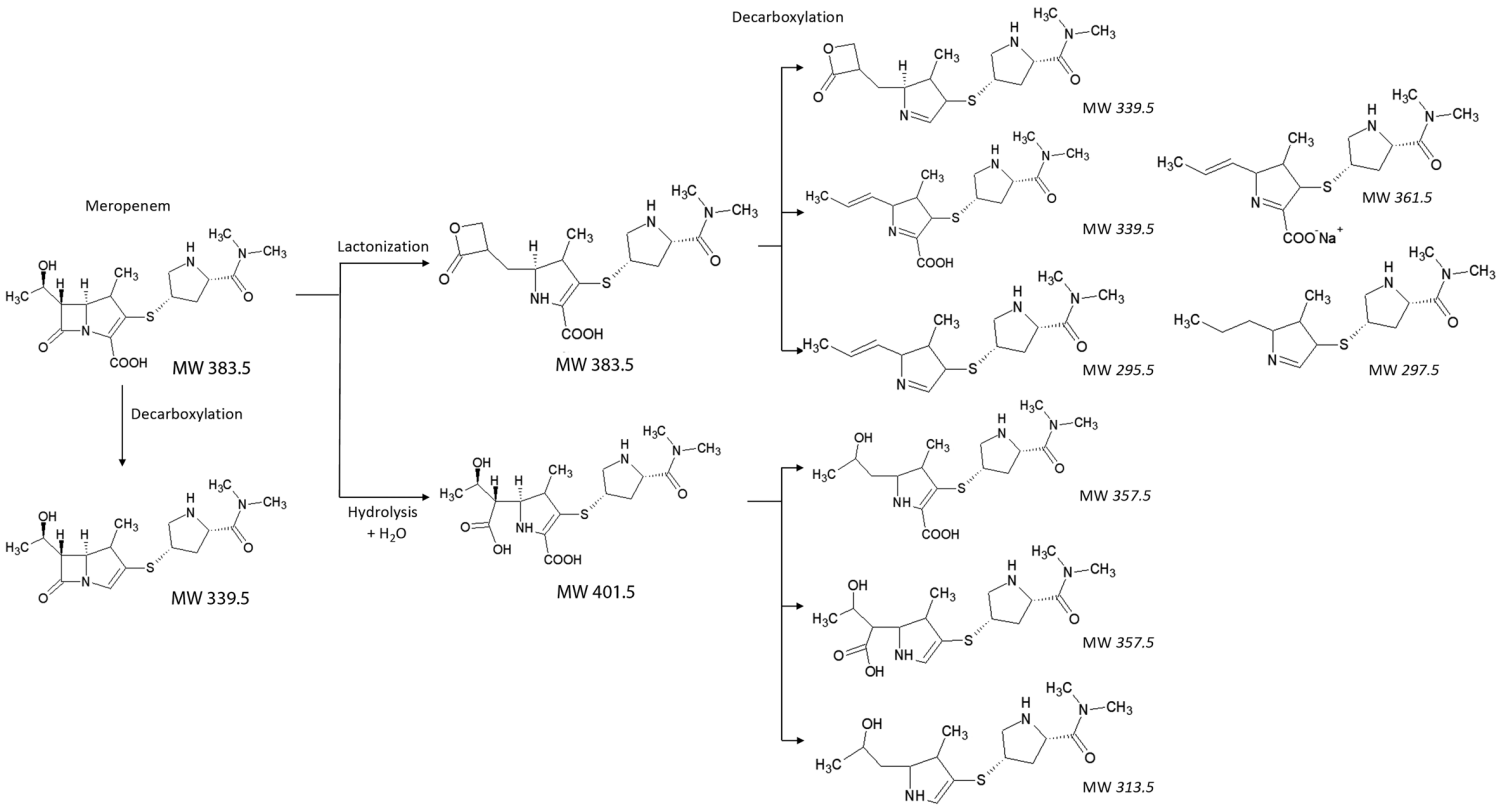
Meropenem, meropenem-derived β-lactone and their decarboxylated molecules with an average molecular weight (MW).

The same experiment was performed with HRAM detection using the FTICR instrument. Figure S4 shows spectra of degradation products due to strain P759 in normal water (Panel A) and in ^18^O labeled water. There is a clear difference by 2 Da, indicating incorporation of water from the environment, which again confirms hydrolytic pathway. Contrary to this, degradation products due to strain P370 are identical in normal and labeled water (Fig. S5). This indicates that isomerization of meropenem due to P370 is an internal rearrangement without outside water being involved (Fig. 3).

### Carbapenemase activity detected by Carba NP and mCIM tests

All the bacteria included in the study were tested using the Carba NP and mCIM tests. Discrepant results were obtained in 5 class A carbapenemase-producing bacteria (GES-5-producing *Enterobacter* spp. and *Escherichia coli*, KPC-3-producing *Klebsiella pneumoniae*) and in no class B carbapenemase-producing strain (see Table 1). Among OXA-48-like producers only, mCIM gave false-negative results in six strains (2 *Escherichia coli*, 3 K*. pneumoniae*. In those strains, a clear hydrolysis of amide-bond of β-lactam ring was observed (presence of a peak at *m/z* 358.5—decarboxylated degradation molecule or its sodium adduct at *m/z* 380.5) as well as β-lactone-corresponding peak at *m/z* 362.5. For Carba NP test, 15 of 35 OXA-48-like-producing strains were interpreted as negative by the technicians as well as by spectrophotometric method (Table 2). Among them, fourteen of those strains degraded meropenem mostly by lactonization with no significant peaks representing hydrolysis of β-lactam ring. No statistical significance was detected by comparison of MALDI-TOF MS analysis and mCIM (p = 0.1091). Presence of β-lactone-corresponding peak at *m/z* 362.5 significantly correlates with negative results of Carba NP test (p = 0.0010).

**Table 2:** Comparison of methods used for a detection of bacteria producing OXA-48-like carbapenemase.

MALDI-TOF MS		mCIM		Carba NP	
		Positive	Negative	Positive	Negative
β-lactone (*m/z* 362.5)	Positive	19	6	10	15
	Negative	10	0	10	0

All 35 isolates were positive by carbapenemase activity detection using MALDI-TOF mass spectrometry (presence of the peak representing decarboxylated hydrolyzed product or its sodium adduct—*m/z* 358.5 and 380.5 and/or β-lactone-corresponding peak—*m/z* 362.5). Detailed characteristics of the isolates are described in Table 1.

## Discussion

Comparing with most common serine carbapenemases (KPC) and metallo-β-lactamases (NDM, VIM) identified in *Enterobacterales*, firstly described OXA-48-like enzymes do not cause resistance to third and fourth generation cephalosporins^30^. Some variants (e.g., OXA-163), however, efficiently hydrolyze broad-spectrum cephalosporins or weakly hydrolyze carbapenems (e.g., OXA-232)^31,32^.

The MICs of carbapenems in OXA-48-like-producing bacteria usually range from susceptible to resistant categories, according to the EUCAST criteria^2,15,17^. The genes encoding for OXA-48-like carbapenemases are usually carried on highly conjugative IncL plasmid enhancing their inter- and intraspecies spread within *Enterobacterales* in bacterial populations, including the microbiota of the same patient^17^. Therefore, the susceptible bacteria possessing OXA-48-like carbapenemase represent substantial epidemiological risk as they can silently spread in the environment. Thus, efficient detection and identification of those enzymes is challenging.

Currently, the most used pH-based direct assays for carbapenemase activity detection fail in the identification of some OXA-48-like carbapenemases^33^. The method, originally described by Skinner and Wise in 1977^34^ for detection of β-lactamases produced by *Haemophilus influenzae*, detects acidification of the reaction mixture after β-lactam ring hydrolysis. During lactonization, however, the pH of the reaction mixture remains unaffected. Even though imipenem does not form β-lactone^10,11^, the strains with preferential lactonization of meropenem were identified as false negative using Carba NP test, presumably due to a low hydrolytic efficiency. On the other hand, one false negative result of mCIM method was identified in the strain clearly producing β-lactone. Those results can further demonstrate antibacterial activity of meropenem-derived β-lactone described elsewhere^10^.

To our knowledge, formation of β-lactone during an infection caused by OXA-48-like producing bacteria has not been studied yet. Based on previously published data^29^, clinical success and survival rates ranging between 0 and 66% in patients treated by meropenem or imipenem monotherapies. For such clinical studies, the strains should be characterized for their ability to inactivate carbapenems by a classical hydrolysis or β-lactone formation. It would be also important to monitor a serum concentration of carbapenems and its degradation products during the therapy^36^.

For the OXA-48 hydrolytic activity, carbamylation of residue Lys-73 residue is essential for activation of a water molecule to deacylation of acyl-enzyme complex^37,38^. Golemi et al*.*^38^ demonstrated that carbamylation of active-site Lys-70 can be influenced by the presence of carbon dioxide in a reaction mixture or by a pH changes with reversible de-carbamylation and loss of the enzymatic activity by exposing the β-lactamase to pH 4.5. The active-site hydrophobic residues also impact the β-lactone synthesis level compared with classical hydrolysis^11^. As we previously demonstrated, addition of bicarbonates can significantly influence MIC value and hydrolytic activity in OXA-48 producing bacteria^12^. Aertker et al.^11^, however, found active site lysine carbamylation has no effect on β-lactone formation when treated with the enzyme by NaH^13^CO_3_. After addition of NH_4_HCO_3_ to the reaction buffer according to the methods previously published^12^, we also detected no changes in β-lactone formation using MALDI-TOF MS and LC–MS respectively.

Based on our results, we propose that the β-lactone formation by OXA-48-like carbapenemases can be detected by LC–MS as an isomeric molecule (*m/z* 384.2) in different retention time compared with the native meropenem molecule. Unlike MALDI-TOF MS, the LC–MS equipment is not widely available in the diagnostic microbiological laboratories. Methods for carbapenemase detection by MALDI-TOF MS, firstly published in 2011^5,6^, are already commercialized as CE-IVD kit^39^. In this study, we have shown that a specific peak representing a decarboxylated sodium adduct of meropenem derived β-lactone can be detected as a peak at *m/z* 362.5, specific for OXA-48-like carbapenemases. Interestingly, the respective peak was already observed in our previously published study^26^ in all OXA-48 (n = 6) and OXA-162 (n = 4) producers across different species (e.g., *Citrobacter freundii*, *Escherichia coli*, *Klebsiella pneumoniae, Raoultella ornithinolytica*). Similarly, any signal was not detected in that area in other non-OXA-48-like carbapenemase-producing bacteria (n = 100) nor in the control strains resistant to carbapenems (n = 35)^26^. For identification of that peak, precise calibration of the mass spectrometer is crucial as the peaks at the *m/z* 358.5 are identified in carbapenemase producers. Proper setting of mass spectrometer (i.e., ion source voltage, detector gain, laser intensity) used in microbiological diagnostics allows obtaining of narrow peaks with excellent differentiation within 1 Da (unpublished data). Another potential marker a peak representing the decarboxylated product β-lactone (*m/z* 340.5) should be verified by automatic software followed by statistical analysis because the peak represents meropenem molecule decarboxylated at the position 3 as unexpectedly detected using isotopically modified water H_2_^18^O (see Fig. 3).

Carbapenem-derived β-lactone formation represents a newly discovered mechanism of carbapenems' inactivation by class D carbapenemases. Deep understanding of the process of lactonization may help in the development of methods for carbapenemase detection. In this study, we have shown differences in the β-lactone formation by OXA-48-producing *Enterobacterales* that can explain low sensitivity of some direct methods used for carbapenemase detection (colorimetric pH-based and MS-based). We also proposed efficient detection of OXA-48-like carbapenemase activity by MS-based techniques.

## Supplementary Information

Supplementary Information 1.

Supplementary Information 2.

Supplementary Information 3.

Supplementary Information 4.

Supplementary Information 5.

Supplementary Information 6.

## Data Availability

The representative spectra are available as supplementary material. All spectra, including raw data, are available upon request from the corresponding author.
