# Supplementary figures and images for: Preferred β-lactone synthesis can explain high rate of false-negative results in the detection of OXA-48-like carbapenemases
Source: Sci Rep. 2022 Dec 23;12:22235. doi: 10.1038/s41598-022-26735-5 (PMC9789108; doi:10.1038/s41598-022-26735-5)

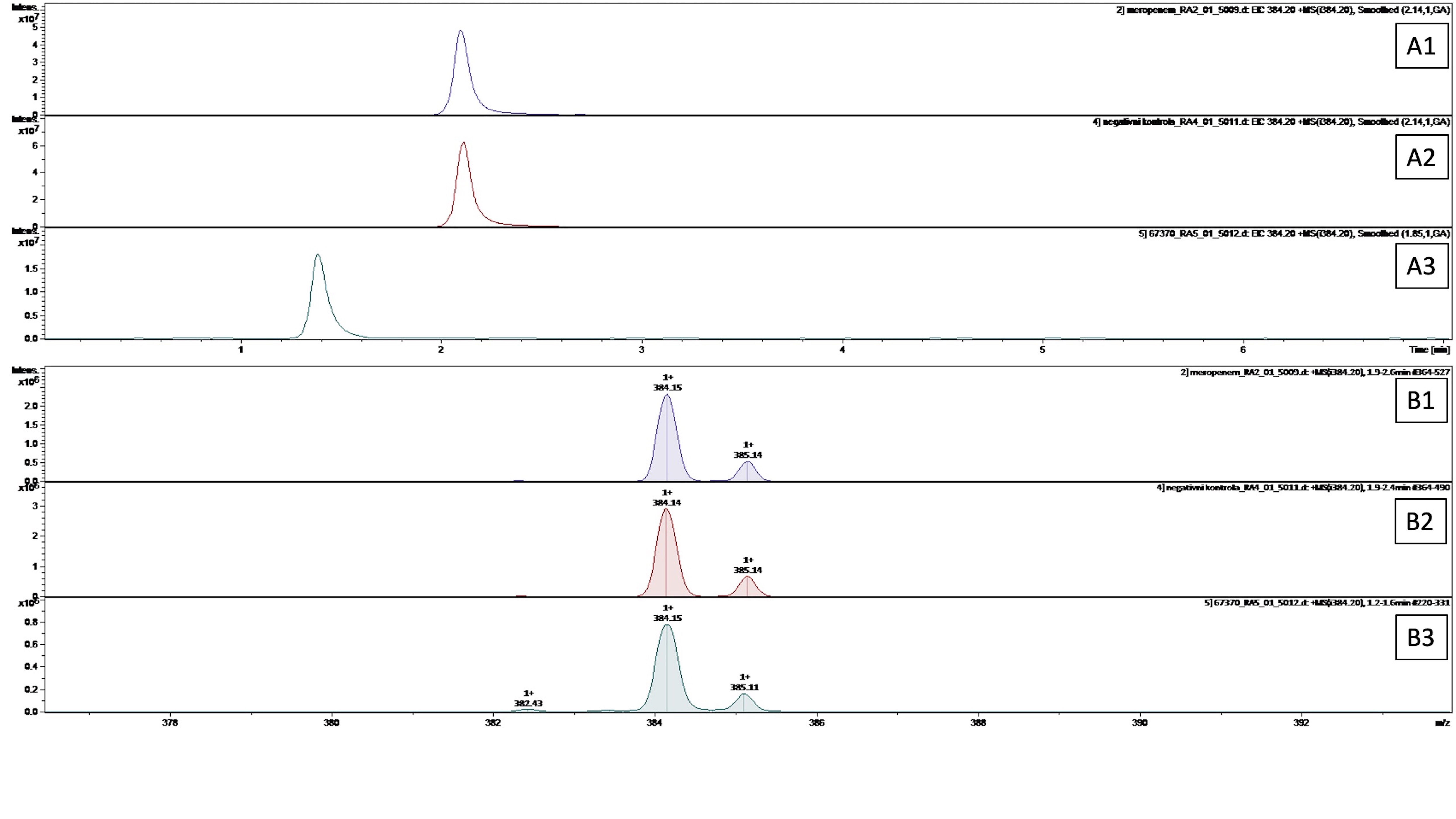

Supplement: Supplementary file 1 — Supplementary Information 1. [file 41598_2022_26735_MOESM1_ESM.jpg]

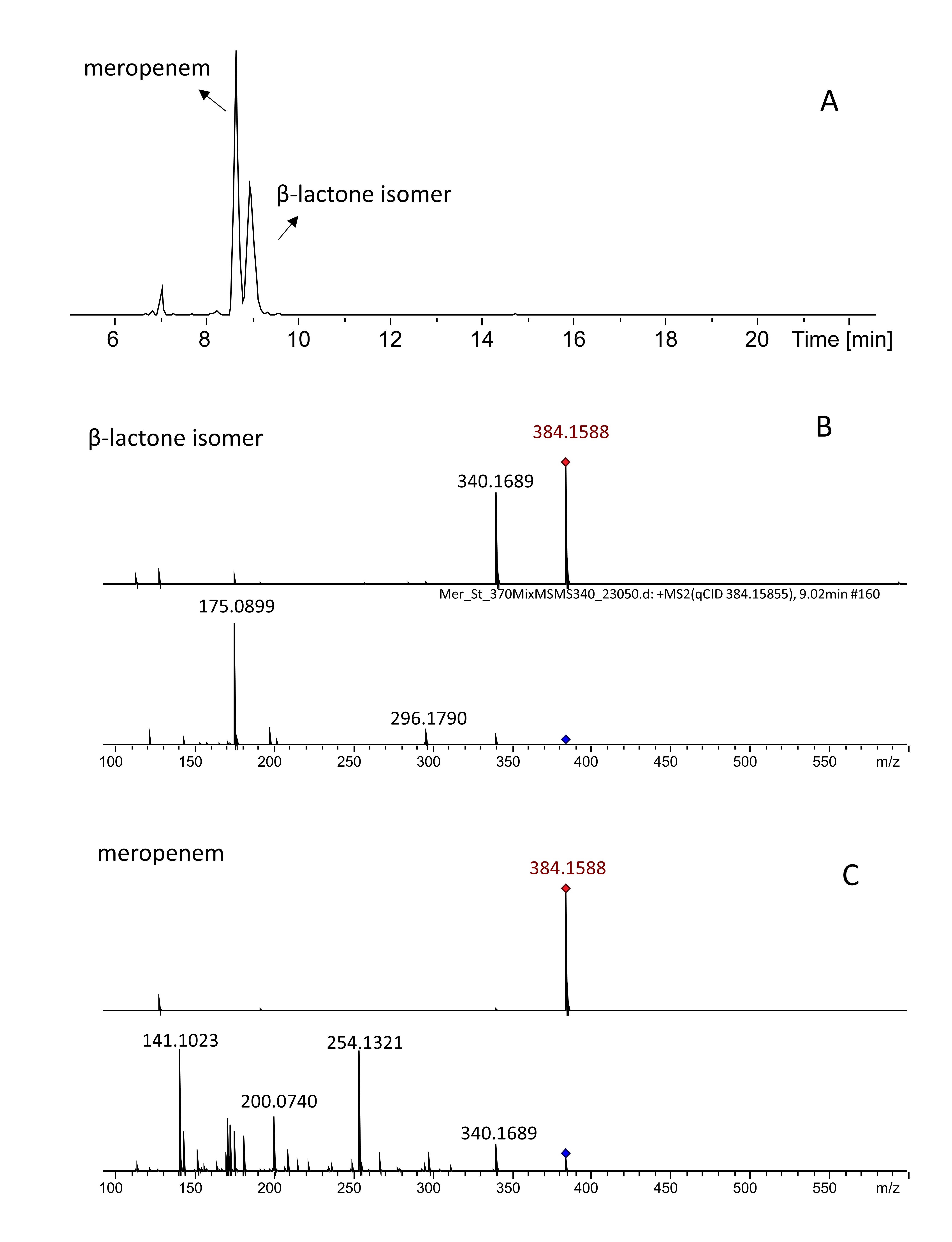

Supplement: Supplementary file 2 — Supplementary Information 2. [file 41598_2022_26735_MOESM2_ESM.jpg]

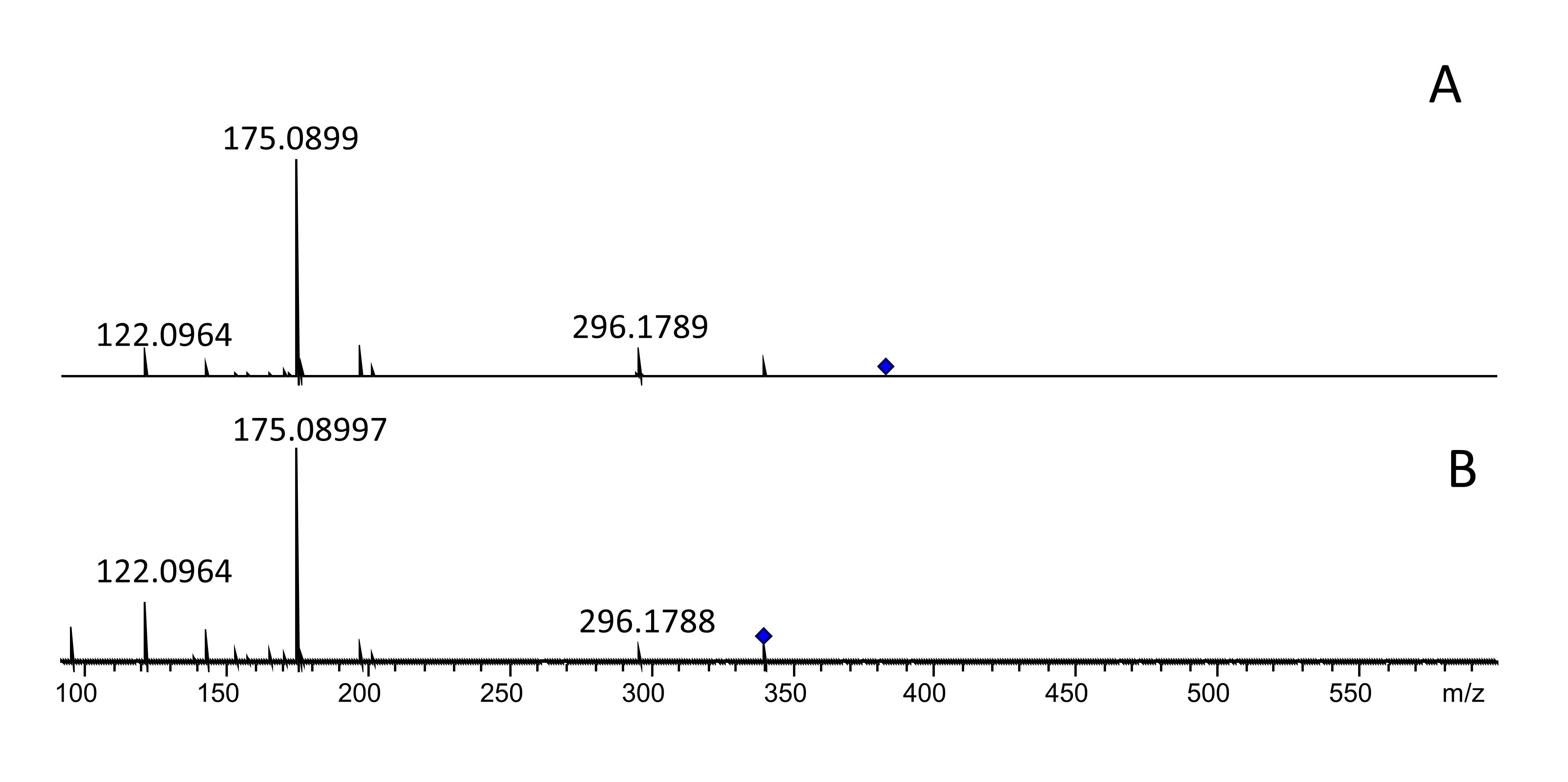

Supplement: Supplementary file 3 — Supplementary Information 3. [file 41598_2022_26735_MOESM3_ESM.jpg]

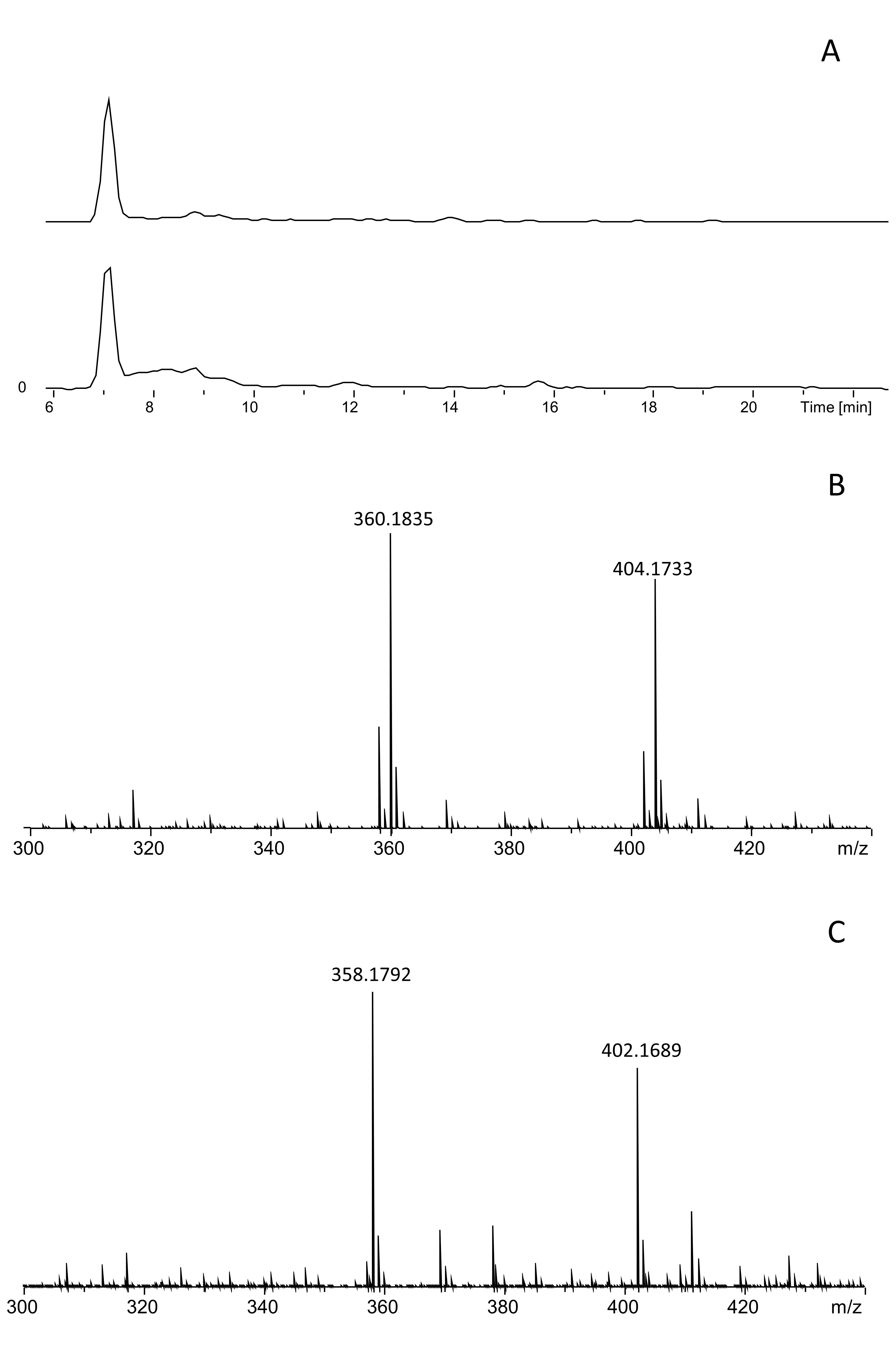

Supplement: Supplementary file 4 — Supplementary Information 4. [file 41598_2022_26735_MOESM4_ESM.jpg]

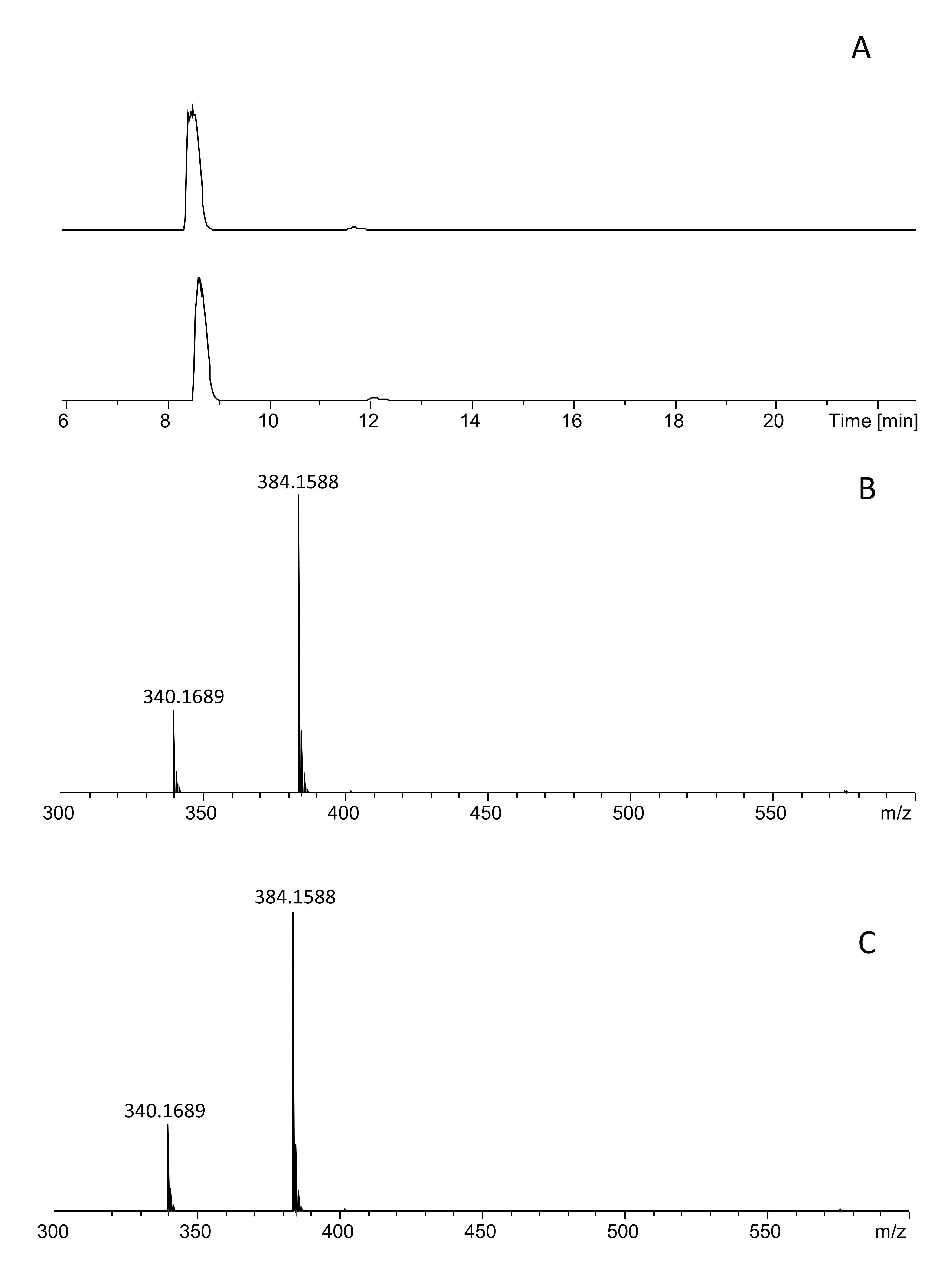

Supplement: Supplementary file 5 — Supplementary Information 5. [file 41598_2022_26735_MOESM5_ESM.jpg]
