# Supplementary material for: Preferred β-lactone synthesis can explain high rate of false-negative results in the detection of OXA-48-like carbapenemases
Source: Sci Rep. 2022 Dec 23;12:22235. doi: 10.1038/s41598-022-26735-5 (PMC9789108; doi:10.1038/s41598-022-26735-5)
Supplement: Supplementary file 6 — Supplementary Information 6. [file 41598_2022_26735_MOESM6_ESM.docx]

**SUPPLEMENTARY FIGURES**

**Figure S1.** LC-MS analysis of the strains P370 (panels A3 and B3) with extracted m/z=384.2 showing differences in the retention time for meropenem (2.1 min.) and meropenem-derived β-lactone (1.4 min.). Panels A1, A2 and B1, B2 represent native meropenem and the negative control respectively (*Escherichia coli* ATCC 14169). Panels A represent chromatograms, panels B represents mass spectra.

**Figure S2.** MSMS spectra of meropenem and its converted isomer. Panel A: Chromatogram of fully converted meropenem with original meropenem spiked back. The chromatographic behavior is identical with the partially converted sample in Figure 2, which confirms identification of both species. Panel B: MSMS spectrum of converted meropenem isomer. Top panel shows precursor selection, the bottom panel shows the MSMS spectrum. Panel C: MSMS spectrum of spiked meropenem standard. Top panel shows precursor selection, the bottom panel shows the MSMS spectrum.

**Figure S3**. MSMS spectra of converted isomer at m/z=384 and the co-eluting species at m/z=340. Panel A: precursor 384; Panel B: Precursor 340

**Figure S4.** Meropenem degradation by strain P759 performed in normal and in ^18^O labeled water. Panel A shows a chromatogram of both samples (top ^18^O labeled, bottom normal water). Panel B shows mass spectrum of degradation products in normal water while Panel C shows the degradation products in ^18^O labeled water. There is a clear shift by 2Da, which indicates incorporation of water from the media into the degradation products, confirming hydrolysis hypothesis.

**Figure S5.** Meropenem degradation by strain P370 performed in normal and in ^18^O labeled water. Panel A shows chromatogram of both samples (top ^18^O labeled, bottom normal water). Panel B shows mass spectrum of degradation products in normal water while Panel C shows the degradation products in ^18^O labeled water. There is no mass difference, which indicates that isomerization of meropenem due to P370 is an internal rearrangement without water from the media being involved.
